# Cures for Pneumonia

**Published:** 1905-12-02

**Authors:** 


### CURES FOR PNEUMONIA.

The writer of an editorial in a recent issue of an American journal [1] falls foul of the dictum of Osler that "Pneumonia is a self-limited disease which can neither be aborted nor cut short by any known means at our command." "Reading this," says the writer, "one need not be surprised to learn from an article in the *American Journal of the Medical Sciences* for June of the present year that 533 cases of pneumonia out of a total of 991 treated in the hospitals of Philadelphia terminated fatally. . . . Is this dreadful waste of human life inevitable, or is it a direct result of the nihilistic teaching of authorities who are grounded in the doctrine of 'self-limited' diseases, etc." In order to prove that the responsibility rests upon the latter the writer has collected an interesting bundle of vaunted cures for pneumonia, which we reproduce, without comment, for the service of those who decline to accept Osler's resigned and passive attitude.

1. Bleeding. This is stated to have been the accepted treatment of pneumonia during the first half of the last century, at which time it would appear from the imperfect statistics available that pneumonia was less fatal than it now is.
2. A single large dose of calomel, 20 to 30 grains or more.
3. Diaphoresis. "Very many country practitioners who do not lose many of their patients from pneumonia, rely almost as a routine measure upon diaphoresis. The patient is put to bed surrounded by hot bottles, and frankly sweated day and night until the disease subsides, which it often does without waiting for the classical crisis."
4. Cold-pack. Dr. Baruch reports only two deaths in more than one hundred cases.
5. Chloroform inhalations. A mortality of no more than 3.5 per cent. has been reported in connection with this treatment.
6. Salicylate of soda.
7. Creosote, either in the form of inhalations, or internally as the carbonate.
8. Potassium iodide in large doses.
9. Quinine in large doses. Reports have been published detailing fifty cases (presumably consecutive) without a death, quinine being the drug employed. One authority is quoted to the effect that a man "with all the signs of pneumonia" took by mistake 60 or 80 grains of quinine in the course of six or eight hours, and promptly recovered.

We confess to a bias in favour of Professor Osler's opinion, although we have no personal experience of any of these methods of treatment, except in some instances as means to combat particular symptoms. The statements of the authorities are too airy and inexact to promote scientific confidence. None the less the writer of the editorial seems reasonable in repudiating a fatalistic acceptance of the *status quo*. Such an attitude is inimical to progress. Conservative practitioners will no doubt continue to prefer the *vis medicatrix naturæ*; those of greater enterprise may find in this therapeutic armament something which appeals to their therapeutic instinct.

[1] Med. Rec., New York, Oct. 28, 1905.

